# Supplementary material for: Hybrid Films from Blends of Castor Oil and Polycaprolactone Waterborne Polyurethanes
Source: Polymers (Basel). 2022 Oct 13;14(20):4303. doi: 10.3390/polym14204303 (PMC9612392; doi:10.3390/polym14204303)
Supplement: Supplementary file 1 [file polymers-14-04303-s001.zip › polymers-1939411-supplementary.pdf]

Supplementary Information to:

Hybrid Films from Blends of Castor Oil and Polycaprolactone Waterborne Polyurethanes

Gastón Pascual, Mirta I. Aranguren * and Verónica Mucci *

Instituto de Investigaciones en Ciencia y Tecnología de Materiales (INTEMA),
Facultad de Ingeniería, Universidad Nacional de Mar del Plata-CONICET,
7600 Mar del Plata, Argentina
* Correspondence: marangur@fi.mdp.edu.ar (M.I.A.); vmucci@fi.mdp.edu.ar (V.M.)

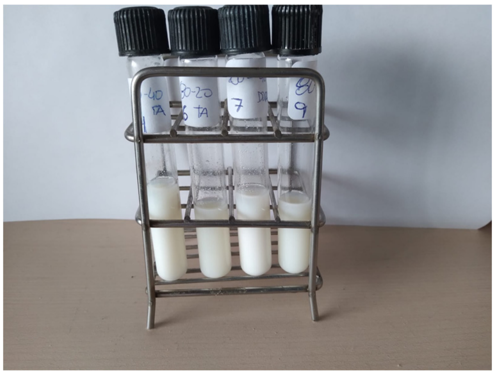

Figure S1. Mixed suspensions in test tubes after 45 days from preparation.

Table S1. Film Thickness

Sample	Thickness (microns)
CO-TA	203.33 ±7.64
PCL-DMPA	296.67 ± 15.28
PCL-TA	253.33 ± 11.55
CO-TA(60%)+ PCL-TA(40%)	250.00 ± 10.00
CO-TA(70%)+ PCL-TA(30%)	336.67 ± 25.17
CO-TA(80%)+ PCL-TA(20%)	250.00 ± 20.00
CO-TA(60%)+ PCL-DMPA(40%)	263.33 ± 15.28
CO-TA(70%)+ PCL-DMPA(30%)	196.67 ± 32.15
CO-TA(80%)+ PCL-DMPA(20%)	336.67 ± 23.09

Table S2. Colorimetric results

Sample	L*	a*	b*	Opacity	WI
CO-TA	86.72±0.70	-4.79±0.7	13.79±3.60	16.89±0.23	80.26±0.63
PCL-DMPA	85.93±0.89	2.43±0.10	-2.37±0.40	31.48±1.50	85.53 ± 0.89
PCL-TA	86.38±1.86	0.77±0.58	-0.67±3.71	27.37±6.60	86.34±1.79
CO-TA(60)/ PCL-TA(40)	88.03±0.30	-0.69±1.57	3.31±1.30	18.93±0.67	87.56±0.28
CO-TA(70)/ PCL-TA(30)	87.29±0.49	-1.81±0.41	5.03±1.97	18.40±0.23	86.21±0.47
CO-TA(80)/ PCL-TA(20)	88.59±0.37	-0.71±0.23	-0.20±0.86	16.90±0.30	88.57±0.36
CO-TA(60)/ PCL-DMPA(40)	88.00±1.37	-0.60±1.83	6.18±4.76	27.16±1.48	86.49±1.24
CO-TA(70)/ PCL-DMPA(30)	87.83±0.18	-0.20±1.11	1.16±0.58	17.50±0.35	87.77±0.17
CO-TA(80)/ PCL-DMPA(20)	84.43±0.56	-3.85±0.27	15.23±1.79	20.37±0.52	77.88±0.54

The analysis to determine statistical differences is shown in the Table S2 (cont.).
The code names for the samples in the tableS2 correspond to:

**M1**	**CO-TA**
**M2**	**PCL-DMPA**
**M3**	**PCL-TA**
**M4**	**CO-TA/PCL- TA (60-40)**
**M5**	**CO-TA/PCL- TA (70-30)**
**M6**	**CO-TA/PCL- TA (80-20)**
**M7**	**CO-TA/PCL-DMPA (60-40)**
**M8**	**CO-TA/PCL-DMPA (60-40)**
**M9**	**CO-TA/PCL-DMPA (60-40)**

For simplicity, when comparing the results obtained from colorimetry, the comparisons between the samples are coded using only the numbers of the name of the samples. Thus, the comparisons between samples M1 and M2 appear in the row with the name "1-2", M1 and M3 as "1-3", and so on.
Colored entries indicate statistically significant difference ($p<0.05$) between the values of the properties of the two samples considered in each row.
Because of the relatively large dispersion of the results for the sample M3 (PCL-TA), probably due to the large particles in the PU dispersion and the crystalline phase, the values measured for this sample do not show (in several cases) clear statistical differences with the other samples. This does not affect the general trend of higher opacity at higher concentration of PCL-based WBPU.

Table S2 (cont)

samples	p (L*)	p (a*)	p (b*)	p (Opacity)	p (WI)
1-2					
1-3					
1-4					
1-5					
1-6					
1-7					
1-8					
1-9					
2-3					
2-4					
2-5					
2-6					
2-7					
2-8					
2-9					
3-4					
3-5					
3-6					
3-7					
3-8					
3-9					
4-5					
4-6					
4-7					
4-8					
4-9					
5-6					
5-7					
5-8					
5-9					
6-7					
6-8					
6-9					
7-8					
7-9					
8-9					

CO-TA

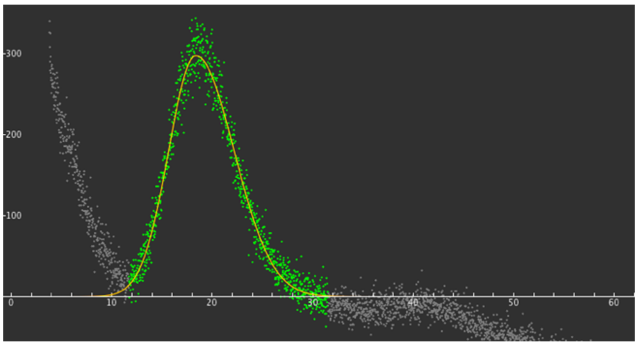

PCL-DMPA

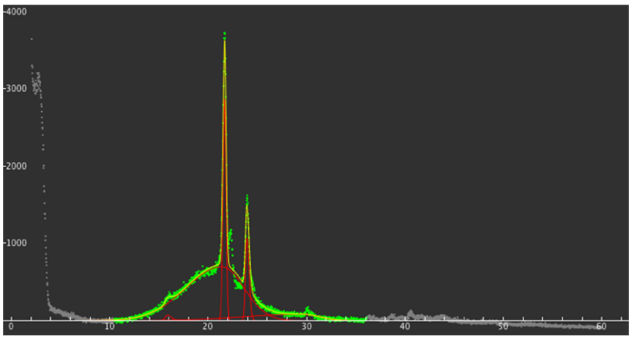

PCL-TA

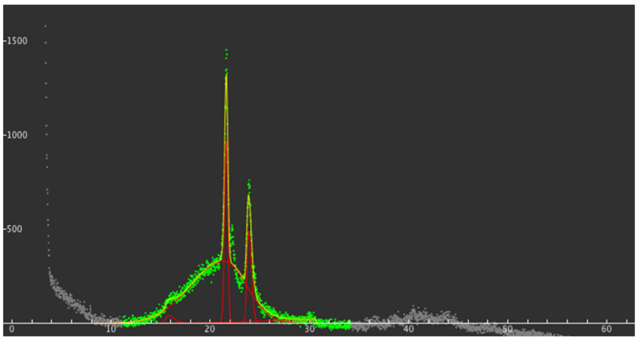

CO-TA(60)/PCL-TA(40)

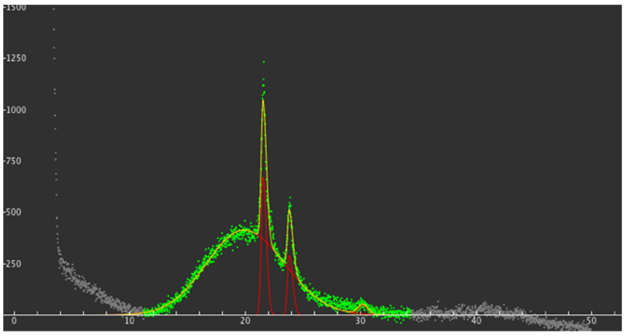

CO-TA(70)/PCL-TA(30)

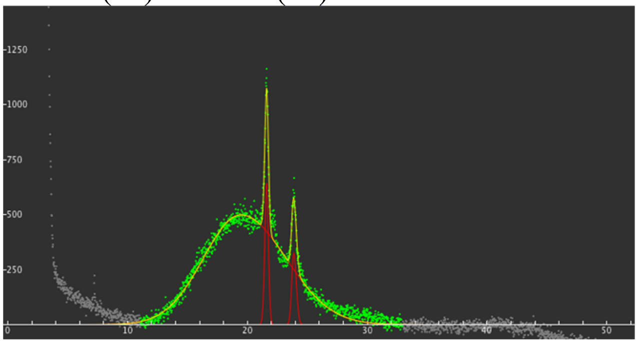

CO-TA(80)/PCL-TA(20)

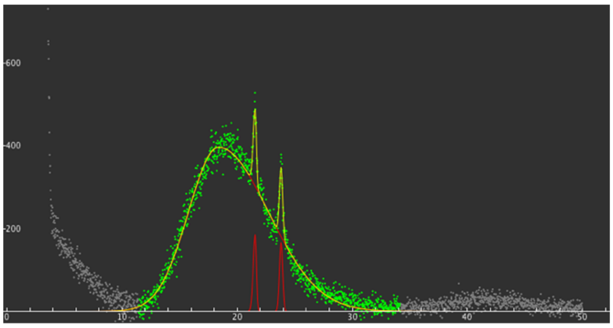

CO-TA(60)/PCL-DMPA(40)

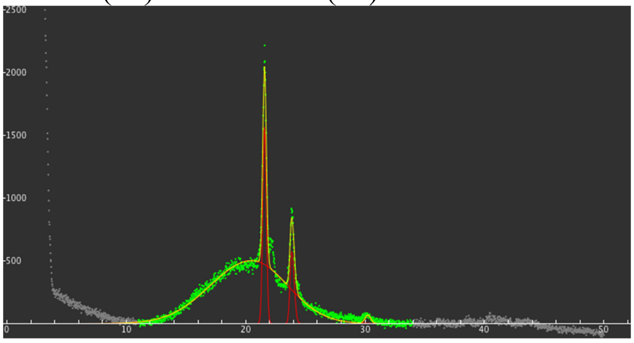

CO-TA(70)/PCL-DMPA(30)

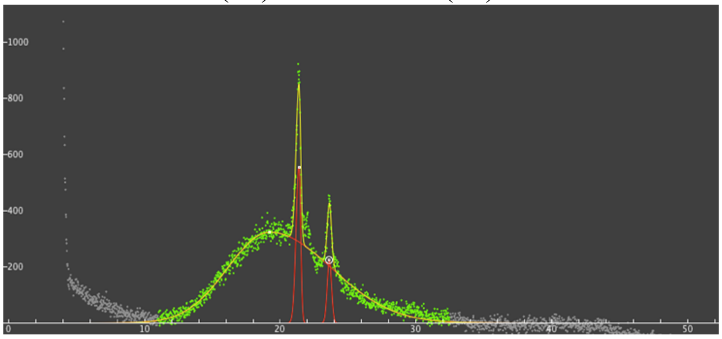

CO-TA(80)/PCL-DMPA(20)

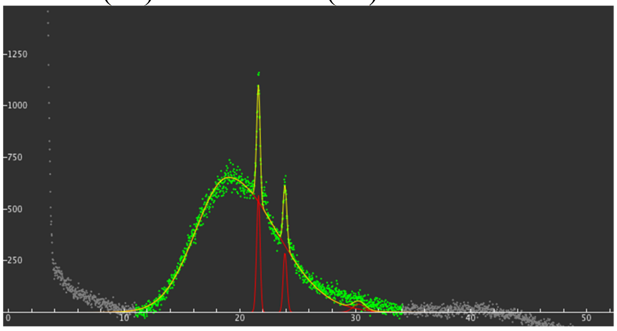

Figure S2 Xray Diffraction-Crystallinity (peak deconvolution)

Table S3. Position and width at half height of the peak at ~21.5°.

**Sample**	**Position**	**Width at half height**
M1= CO-TA	---	---
M2=PCL-DMPA	21.68	0.3638
M3=PCL-TA	21.63	0.3853
M4=CO-TA(60)/ PCL-TA(40)	21.53	0.5163
M5=CO-TA(70)/ PCL-TA(30)	21.60	0.3628
M6=CO-TA(80)/ PCL-TA(20)	21.57	0.3278
M7=CO-TA(60)/ PCL-DMPA(40)	21.58	0.3505
M8=CO-TA(70)/ PCL-DMPA(30)	21.54	0.3358
M9=CO-TA(80)/ PCL-DMPA(20)	21.62	0.3536

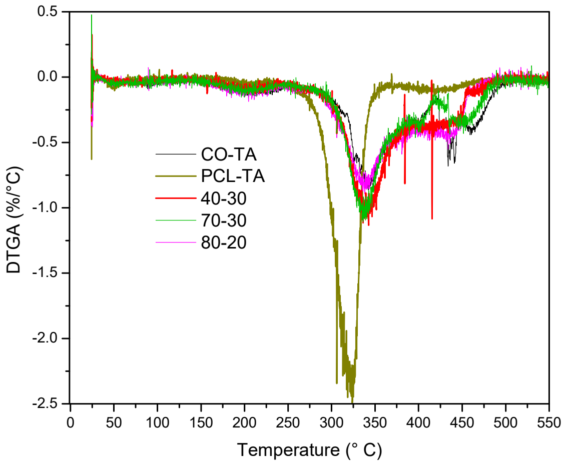

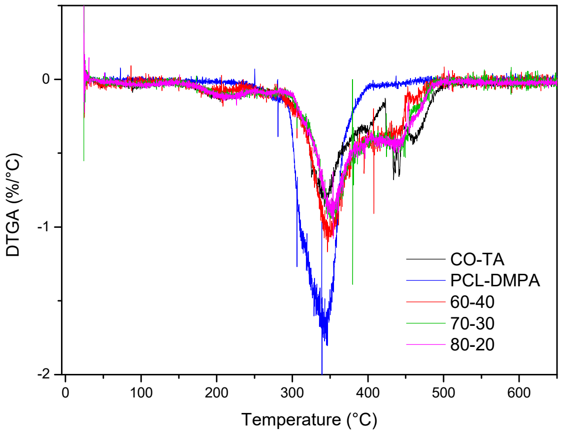

Figure S3. Thermogravimetry: dTG curves

Table S4. Statistical analysis for assay solubility and swelling in toluene.

Sample	% gel	% error gel	% en tol.	% error tol.
CO-TA	93.02[a]	1.45	39.65[a]	2.48
PCL-DMPA	1.92[b]	0.64		
PCL-TA	2.84[b]	0.49		
CO-TA/PCL-TA 60-40	61.2[c]	0.73	38.62[a]	3.52
CO-TA/PCL-TA 70-30	77.08[d]	1.29	44.94[a]	7.23
CO-TA/PCL-TA 80-20	85.7[e]	0.05	77.02[b]	4.26
CO-TA/PCL-DMPA 60-40	59.14[c]	1.22	8.85[c]	0.73
CO-TA/PCL-DMPA 70-30	71.06[f]	1.13	24.31[d]	1.19
CO-TA/PCL-DMPA 80-20	88.480[a,e]	2.6	65.12[e]	0.45

Table S5. Water contact angle with representative images of the measurements.

	Image	Water contact angle
CO-TA	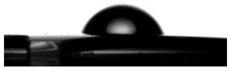	73.14 ± 0.19
PCL-DMPA	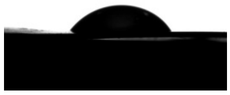	63.39 ± 0.07
PCL-TA	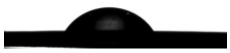	50.19 ± 0.13

CO-TA(60)/PCL-TA(40)	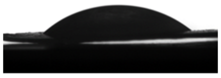	40.1 ± 0.49
CO-TA(70)/PCL-TA(30)	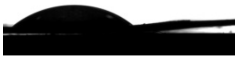	54.20 ± 0.29
CO-TA(80)/PCL-TA(20)	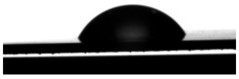	54.98± 0.28
CO-TA(60)/PCL-DMPA(40)	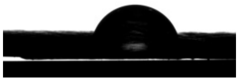	63.69± 0.43
CO-TA(70)/PCL-DMPA(30)	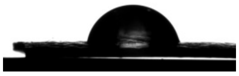	60.8 ± 0.34
CO-TA(80)/PCL-DMPA(20)	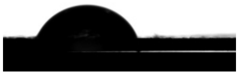	61.28 ± 0.12
